# Supplementary material for: Are carbon emissions trading and green financial instruments synergistic? -Comprehensive quantitative research based on content analysis
Source: PLoS One. 2024 Mar 7;19(3):e0298601. doi: 10.1371/journal.pone.0298601 (PMC10919601; doi:10.1371/journal.pone.0298601)
Supplement: S1 Data — (ZIP) [file pone.0298601.s001.zip › Data/地级市Z_F工作报告.zip!/╔╜╢1⁄2/╠⌐░▓╩╨/└┤╫╘╣1⁄2╓┌║┼í╛┬φ┐╦╩2╛▌═°í┐_2011.docx]

现在，我代表泰安市人民政府向大会报告工作，请连同《泰安市国民经济和社会发展第十二个五年规划纲要(草案)》一并审议，并请市政协委员和其他列席人员提出意见。

　 一、“十一五”期间全市经济社会发展取得显著成就

　 “十一五”时期是泰安发展历程中极不平凡的五年。面对国际金融危机的巨大冲击和国内外复杂环境的风险挑战，全市上下在中共泰安市委的坚强领导下，坚持以邓小平理论和“三个代表”重要思想为指导，认真贯彻落实党的十七大和十七届三中、四中、五中全会精神，以科学发展观统揽全局，按照市委确定的工作思路，解放思想，科学务实，积极作为，顽强拼搏，全市改革开放和现代化建设取得显著成就，圆满完成了“十一五”规划的主要目标任务。

　 经济综合实力显著增强。2010年全市完成生产总值2051.7亿元，比上年增长13.7%，五年年均增长14.7%，其中一二三产业增加值年均增长3.9%、15.1%和17.3%。完成地方财政收入116.9亿元，比上年增长27.9%，年均增长23.5%，比“十五末”增长1.9倍。全市金融机构各项存、贷款余额分别达到1399.5亿元和917.6亿元，年均增长17.1%和16.5%。

　 转方式调结构取得积极进展。三次产业比例由2005年的12.4：55.5：32.1调整为“十一五”末的9.5：53.6：36.9。农业综合生产能力和产业化水平明显提高，粮食生产连续八年实现稳定增产，其他主要农产品产量稳步增长，有机蔬菜、奶牛养殖、苗木花卉、桑蚕生产等成为农业发展的亮点，小麦、玉米高产技术获全国农牧渔业丰收一等奖。工业主导产业、骨干企业和知名品牌培植取得明显成效，八大主导产业调整振兴实现新突破，规模以上工业企业达到1704家，主营业务收入、利税、利润分别达到3904.2亿元、496.0亿元、307.0亿元，年均增长31.1%、32.2%和37.3%；其中主营业务收入过10亿元、实缴税金过亿元的企业达到53家和8家，分别比2005年增加42家和3家。中国名牌产品、中国驰名商标分别达到6个和15件。服务业尤其是旅游文化产业发展迅速。社会消费品零售总额累计完成2458.4亿元，年均增长19.9%；旅游总收入达到253.5亿元，年均增长30.8%，占生产总值的比重由“十五”末的7.6%提高到12.4%。创新型泰安建设全面启动，高新技术产业产值占规模以上工业总产值的比重达到34.2%，比2005年提高12.4个百分点，泰安被列为“国家知识产权示范创建市”，跻身“中国城市综合创新能力50强”。节能减排取得明显成效，万元GDP能耗、规模以上工业万元增加值能耗、二氧化硫和COD排放量持续下降，全面完成省下达的节能减排任务。生态环境明显改善，全市森林覆盖率达到36.9%。

　 项目建设成绩显著。全市规模以上固定资产投资累计完成4255.9亿元，是“十五”投资总量的3.5倍，年均增长23.8%。投资结构逐步优化，其中高新技术产业、三产投资年均分别增长20.9%和30.7%。累计投资建设亿元以上的重点项目687个，投资总规模3443亿元。先后争取中央投资项目229个、4.1亿元，省调控资金项目53个、11亿元。青年汽车、山东煤机工业园、泰山方特欢乐世界、泰山封禅大典实景演出、水浒文化主题公园、精制盐、瑞阳多晶硅、石墨电极、合浩通电缆、东顺工业园等326个过亿元项目建成投入运营；石膏板护面纸、特变电工华东产业园、中联美景400万吨水泥熟料、志高国际广场等259个过亿元项目正在加快建设。水利、交通、通信、能源等基础设施进一步完善，对经济社会持续发展的拉动支撑作用明显增强。

　 改革开放不断深化。市级国有企业改制基本完成，国有资产管理体制改革进一步深化。新增上市企业5家。农村税费改革和综合配套改革不断深化，土地流转制度、集体林权制度改革稳步推进。实施了新一轮市、县、乡政府机构改革，财税、金融、投融资、粮棉流通等体制改革取得实质性进展，教育、文化、医药卫生、社会保障等领域体制改革力度加大。全市新开工总投资3000万元以上的国内招商引资项目2648个，到位资金1008.2亿元，年均增长21.3%；累计新批利用外资项目595个，实际利用外资55.4亿美元，年均增长19.4%；美国马尼托瓦克机械公司、韩国现代重工、日本昭和电缆和中国建材集团、重汽集团、中材集团等国内外一批知名大企业大集团入驻泰安。累计完成进出口贸易总值65亿美元，其中出口40.9亿美元，年均分别增长16.4%和11.2%。对外经济技术合作和境外投资取得新进展。

　 城乡面貌明显提升。按照新一轮城市总体规划，组织编制了各类总体规划和专业规划。全力推进“创城”工作，实施了“两轴”、“两带”、旅游经济开发区、京沪高铁泰安站新区、大汶河与长城路等片区开发，加强基础设施建设、城乡环境综合整治和城中村改造，中心城市的辐射带动作用日益增强。县级城市加快发展，中心城镇、新农村建设取得积极成效。行政村“村村通柏油路、通客车、通有线电视”率分别达到99.7%、99.5%和100%。市高新区建设发展取得新成绩，泰山景区荣膺“世界地质公园”、首批全国文明风景旅游区和首批国家5A级旅游景区等称号。国家卫生城市顺利通过复审，预计全市城镇化率达到50%。泰安被评为“国家历史文化名城”、“国家园林城市”；新泰、肥城被命名为“国家园林城市”。

　 社会更加和谐稳定。城乡免费义务教育全面实现，高中阶段教育基本普及，职业教育、成人教育持续发展。编制实施《泰安市中心城区中小学布局专项规划》，新建和加固校舍面积66.8万平方米，农村中小学办学条件逐步改善。积极支持驻泰高校的改革与发展，在校生达到9.5万人。87个乡镇(街道办事处)全部建成了综合文化站，公共文化服务体系建设扎实推进，文物保护得到加强，大汶口文化遗址列入国家“十二五”大遗址保护规划。城乡医疗卫生体系不断完善，2350所卫生室全部完成新、扩、改建任务，城市社区卫生服务实现全覆盖，新农合参合率达到99.84%。奥运金牌实现零的突破，奥运火炬传递、全运圣火采集和赛事承办圆满成功，公共体育设施建设得到改善，群众体育广泛深入。人口继续保持低生育水平，年均人口自然增长率为4.06‰。生态环境保护力度加大，群众性精神文明创建活动深入开展，民主法制建设得到加强。深化“平安泰安”建设，重视加强信访和社会矛盾化解工作，全面加强社会治安综合治理，切实抓好安全生产和食品药品监管，组建了市政府应急管理平台，应对和处置突发事件的能力明显提升。对口支援、扶贫协作、美国白蛾防治、重大疫病和疫情防控取得积极成果。国防动员、人防建设、民兵预备役、双拥共建等取得新成绩。妇女儿童和残疾人保障、老龄工作及服务有新的提高，外事侨务、民族宗教、爱国卫生、气象、防震、史志、档案等项社会事业全面进步。我市先后荣获“全国双拥模范城”、“全国民族团结进步模范集体”等称号。

　 民生得到持续改善。城镇居民人均可支配收入、农民人均纯收入分别达到19953元和7592元，年均增长14.1%和13.0%。以创建国家级创业型城市为契机，实施全民创业战略，每年城镇新增就业再就业7万人左右，累计转移农村劳动力40余万人，城镇登记失业率控制在3.5%以内。覆盖城乡居民的社会保障体系初步形成，社会保障水平明显提高。五项社会保险实现市级统筹，社会保险收支总规模达到352亿元，各项社会保险费收缴率均达95%以上，企业离退休人员养老金社会化发放率保持100%。城镇居民基本医疗保险全面推开，新型农村社会养老保险试点顺利推进；提高了失业保险、城乡低保、五保供养、新农合补助标准。累计归集住房公积金52.6亿元，发放贷款40.9亿元。建设经济适用住房4359套、廉租住房2514套和公共租赁住房656套，全面建立了廉租房租金补贴制度；321个村启动了整村迁建，建设农房6.6万户，完成危房改造1.9万户。全面完成了12座大中型、411座小型病险水库除险加固任务，农村自来水普及率达到98%。完成农村无害化生态卫生厕所28.6万座，新建户用沼气池8万多个。

　 行政服务能力建设得到加强。深入贯彻《行政监察法》、《公务员法》和国务院《全面推进依法行政实施纲要》等法律法规，健全完善执法责任追究、执法评议考核、行政效能监察和举报投诉等制度，实行涉企检查、收费备案和首次不罚教育警示等制度。全面清理行政许可和非行政许可事项，减少市级行政审批事项117项，集中清理规范性文件、政策性文件1800件，废止573件、修改99件。加强行政服务标准化和公共服务平台建设，开通了便民服务电话、民生直通车网站，电子政务、政务公开、行风评议等深入开展，被评为全国政务公开先进单位。重视加强政府廉政建设和领导干部经济责任审计，大力加强公务员队伍建设，干部队伍的整体素质和施政能力不断提升。

　 各位代表，五年的发展成就鼓舞人心、来之不易，成功经验需要继承发扬、丰富完善，主要是：必须坚持以科学发展观为指导，认真贯彻执行党的基本路线、方针、政策和一系列重大决策部署，坚持以经济建设为中心不动摇，处理好保增长、转方式、惠民生的关系，坚定不移地推进科学发展；必须坚持不断解放思想，提升思想境界和工作标准，敢于创先争优，勇于创新体制机制，不断扩大对外开放，努力推动跨越发展；必须坚持求真务实、真抓实干，努力做好上级指示精神与泰安实际相结合的文章，想干事、敢干事、会干事、干成事；必须坚持统筹兼顾，促进产业、区域、城乡、人与环境协调发展，推动经济建设、社会建设、文化建设、生态文明建设相互促进繁荣发展；必须坚持以人为本，把改善民生、促进人的全面发展作为出发点和落脚点，真心实意地为群众办实事，让人民群众共享改革发展成果。

　 各位代表，五年来我市经济与社会发展取得的成绩，是市委坚强领导，市人大、市政协及社会各界有效监督和大力支持的结果，是全市人民解放思想、争先进位、团结拼搏的结果，也是驻泰各单位和各界人士全力支持、自觉融入泰安经济和社会发展的结果。在此，我代表泰安市人民政府，向全市广大干部群众、各民主党派、工商联、无党派爱国人士，向人民解放军和武警驻泰部队及驻泰单位，向关心支持泰安发展的港澳台同胞、海外侨胞及海内外朋友，表示崇高的敬意和衷心的感谢！

　 在充分肯定成绩的同时，我们也清醒地看到，经济社会发展中还存在不少困难和问题，政府工作也有许多不足。主要是：经济总量仍然偏小，经济结构不够合理，工业主导产业优势不明显，高新技术产业比较薄弱，骨干企业较少、核心竞争力不强，服务业比重偏低，与我市独特的区位资源优势特别是建设国际旅游名城的定位还不相称；经济发展的质量和效益不够高，财政收入占生产总值的比重、主体税收占财政收入的比重偏低，自主创新能力不够强，经济增长的资源、环境约束加大，节能减排压力较大，转变经济发展方式的任务非常艰巨；改革开放的力度需要进一步加大，体制性、机制性障碍依然存在，外向型经济比较滞后；城市功能还需要进一步完善，城乡统筹任务艰巨；社会建设和管理还存在很多薄弱环节，保障和改善民生任务繁重，公共服务水平有待进一步提高；政府工作人员的思想观念需要进一步解放和更新，作风有待进一步转变，行政效能和服务质量需要进一步提高等等。对此，我们将采取更加有力的措施，认真加以解决。

　 二、“十二五”时期的总体要求和目标任务

　 “十二五”时期是我们把握重要战略机遇期、在新的历史起点上加快强市名城建设、全面建设小康社会的关键时期。政府工作的总体要求是：高举中国特色社会主义伟大旗帜，以邓小平理论和“三个代表”重要思想为指导，深入贯彻落实科学发展观，按照市委确定的“一二三四五”工作思路，紧紧围绕科学发展这个主题，牢牢把握转方式调结构这条主线，坚定不移地以强市名城建设为奋斗目标，深化改革开放，保障和改善民生，促进经济长期平稳较快发展和社会和谐稳定，为全面建成小康社会打下具有决定性意义的基础。经过五年的努力，使综合实力显著提升，经济结构明显优化，城乡区域协调发展，社会事业繁荣兴盛，人民生活殷实富裕，生态环境优美宜居。到2015年，全市生产总值突破3400亿元，年均增长11%；地方财政收入达到235亿元，年均增长15%；三次产业比例调整为7：48：45；全社会固定资产投资、社会消费品零售额分别达到2900亿元和1550亿元，年均增长18%，进出口总值五年累计突破120亿美元，年均增长15%以上；全市城镇化水平达到56%；城镇居民人均可支配收入和农民人均纯收入年均增长11%，累计新增就业再就业25万人，城镇登记失业率控制在3.5%以内；单位GDP能耗、水耗、污染物排放量等资源环境约束性指标达到或好于全省要求，森林覆盖率达到39.5%。

　 实现上述目标，我们既面临着国际和区域间竞争更趋激烈、传统产业转型、资源环境制约等诸多可以预见和难以预见的风险挑战，更面临着难得的历史机遇。全球经济正在进入后国际金融危机时代；我国经济发展长期向好的趋势没有改变，国家实施积极的财政政策和稳健的货币政策，为经济稳定增长提供了保障；我市面临着国内经济持续增长、承接国内外产业调整转移、省会城市群经济圈加速推进、交通区位优势更加明显等难得的历史机遇，特别是经过几次解放思想大讨论，全市干部群众加快发展、干事创业、争先进位的热情和积极性更加高涨、氛围更加浓厚、干劲更加充足。只要我们牢牢抓住重要战略机遇期，坚定信心，因势利导，科学务实，顽强拼搏，就一定能够圆满完成“十二五”规划的各项任务目标。在工作的指导和把握上，要做到“六个坚持”：即坚持转型发展，把转方式调结构作为重大任务和主攻方向，构建扩大内需长效机制，积极扩大消费需求，合理扩大投资规模，调整优化投资结构，努力扩大出口，促进经济增长向依靠消费投资出口协调拉动、一二三产业协同带动转变，促进经济向提高效益、节约资源转变。

　 坚持创新发展，把改革贯穿于经济社会发展的各个方面，加快推进重点领域和关键环节的改革，推进体制机制创新和工作创新，深入实施人才强市和科教兴泰战略，培育创新主体，打造创新平台，优化创新环境，激发创造活力。坚持统筹发展，把统筹兼顾作为科学发展的根本方法，统筹经济与社会、城市与农村、当前与长远，加快实施重点区域带动战略，积极稳妥推进城镇化，加快推进社会主义新农村建设，促进区域城乡协调发展，提高发展的全面性、协调性和可持续性。坚持绿色发展，把建设资源节约型、环境友好型社会作为加快转变经济发展方式的重要着力点，深入实施可持续发展战略，加大节能减排和环境保护力度，大力发展循环经济、绿色经济、低碳经济，增强可持续发展能力，提升人居环境。坚持开放发展，把对外开放作为加快转变经济发展方式的强大动力，实施更加积极主动的开放战略，推进对外经济文化交流，积极主动地参与区域合作，努力提升开放型经济和先进文化对发展的拉动作用，增强发展的融合性和开放性，以开放促发展、促改革、促创新。坚持和谐发展，把保障和改善民生作为根本出发点和落脚点，把推进就业放在优先位置，提高教育医疗服务水平，健全完善社会管理和保障体系，推进基本公共服务均等化，促进社会公平正义，确保社会稳定，使社会成员都能充分享受改革发展的成果，不断满足人民群众过上更好生活的新期待，更加充分地激发和调动广大干部群众的积极性和创造性。

　 三、突出重点，强力推进，确保今年经济与社会平稳较快发展

　 实施“十二五”规划，五年看头年，做好今年的工作至关重要。按照市委的总体部署安排，今年全市经济社会发展的主要预期目标为：全市生产总值增长12%左右；地方财政收入增长16%；规模以上固定资产投资增长20%左右；社会消费品零售总额增长18%左右；外贸进出口增长15%左右，实际利用外资增长20%左右；城镇居民人均可支配收入和农民人均纯收入均增长12%；城镇新增就业再就业5.5万人，城镇登记失业率控制在3.5%以内，人口自然增长率控制在7.8‰以内;居民消费价格涨幅控制在4%左右；全面完成省政府下达的万元生产总值能耗和二氧化碳排放强度、化学需氧量、二氧化硫、氮氧化物和氨氮排放量等节能减排任务。在实际工作中，力求发展得更好一些、更快一些。

　 (一)坚持强农惠农，大力推进现代农业建设。按照在工业化、城镇化深入发展中同步推进农业现代化的要求，夯实农业农村发展基础，着力促进农民增收。一是加快推进农业结构优化升级。深入开展优质粮食高产创建活动，大力发展高效特色农业，实施有机蔬菜、奶牛养殖、花卉苗木、茶叶、蚕茧等14个农业产业振兴规划，构筑特色优势农业生产体系。二是提升农业产业化经营水平。加大对农业龙头企业的扶持力度，着力培植一批产业关联度高、带动能力强、有较强竞争力的大中型农产品加工企业，推进农产品加工产业聚集和龙头企业集群发展，年内力争全市规模以上农业龙头企业达到400家。抓好农民专业合作组织发展和农村市场体系建设，推进农产品区域化管理、农超对接等工作，确保农产品质量安全。三是强化农业发展基础支撑。抓住国家启动新一轮水利设施建设的机遇，搞好农田水利重点县、河道治理等工程建设。搞好小流域综合治理，实施农村饮水安全工程，年内解决10万人的饮水安全问题。认真贯彻落实《山东省用水总量控制管理办法》，建立最严格的水资源管理制度。进一步搞好中低产田改造，大规模建设旱涝保收基本农田。实施农机创新示范工程，提高农业生产装备水平。推进农村土地综合整治、农业综合开发和农村道路建设，不断改善农村生产生活条件。四是进一步拓宽农民增收渠道。全面落实好国家粮食直补、农资综合补贴、良种补贴、农机购置补贴等各项强农惠农政策，增加农民转移性收入。鼓励支持农民优化种养结构，增加生产经营性收入。加快发展农村二、三产业，在农民职业技能培训、贷款发放、税费优惠等方面搞好服务，促进农民就地就业和转移就业，增加农民工资性收入。坚持抓好农民负担监督管理，防止农民负担反弹。

　 (二)坚持转调并重，促进工业做大做强。以传统产业改造升级、重点产业培植增强、产业集群和特色产业聚集壮大、新兴产业加快发展为切入点，积极构建以传统产业为基础、以优势产业为龙头、以新兴产业为重要增长点的高端高质高效产业体系，创造产业竞争新优势。一是培强做大优势产业和骨干企业。把先进制造业作为工业经济发展的重要着力点，继续实施八大产业调整振兴规划，发挥龙头企业的带动作用，迅速扩张规模，延伸产业链条，着力培植汽车、输变电设备、无机非金属材料、装备制造等优势产业，打造 10个特色产业集群，培育一批规模大、效益好、市场竞争力强的骨干企业，重点打造过百亿的大企业集团。大力推进中小企业成长计划和中小企业培育计划，搞好信贷担保服务，积极为中小企业发展搭建平台、创造条件。二是加快改造提升传统产业。以“四个一百”项目为抓手，采用高新技术和先进适用技术改造提升煤炭、化工、纺织、食品等传统产业，推动信息化和工业化深度融合，增强新产品研发能力和品牌创建能力，以先进产能淘汰落后产能，促进传统产业加速优化升级，进一步提高综合竞争力。三是大力发展战略性新兴产业。围绕新材料、新能源与节能环保、电子信息、生物医药与生物育种等重点领域，强化技术和产品开发、产业化、市场培育等关键环节，扶持重点产业和骨干企业发展。研究制定高端产业发展规划及扶持政策，集中打造“一带四片”高端产业发展新格局，带动工业向高端高质高效发展。加快高新区等各类园区发展，使其成为高端高质高效产业发展的先行区、聚集区、示范区。四是精心组织搞好工业经济运行。强化对工业运行的监测分析，及时发现和解决经济运行中的苗头性、趋势性问题，加强对能源、原材料、资金、运力等组织协调，着力提高要素保障能力，保持工业经济平稳较快增长。深入开展“市场营销年”活动，引导企业抢抓机遇，全力开拓市场，努力扩大产品市场占有份额。

　 (三)坚持发挥特色，推动服务业跨越发展。把服务业作为经济结构调整的战略重点和主要经济增长点，深入实施《泰安市服务业发展规划纲要(2010—2020)》，认真抓好各项政策措施的落实，不断提升服务业发展的规模和档次，努力提高服务业占生产总值的比重和对财政增长的贡献率。一是突出发展旅游文化产业。充分发挥“山水圣人”旅游线优势，以打造山东旅游文化产业高地为目标，深入实施“融合济南、辐射全省、对接京沪、面向全国、拓展海外、放眼世界”的旅游发展战略，整合资源，深度融合，加快主体培育和项目建设，优化旅游服务，努力提升旅游文化产业的水平和档次。以泰山为龙头，以泰城为主体，整合大汶口文化遗址、徂徕山、莲花山、肥城桃园、宁阳神童山、东平湖等旅游资源，集中打造泰山观光、城市休闲度假、山水特色体验等旅游产品集群，加快构建泰安大旅游产业圈。培育创意旅游项目，开发高端特色旅游产品，加强旅游业与其他服务业的互动发展，延伸旅游产业链条。强化旅游载体和文化建设，打造泰山文化、大汶口文化、水浒文化等特色文化品牌，大力发展各种文化旅游，做好“吸引人、留住人”的文章。二是加快发展现代服务业。继续加快实施服务业载体建设“103030工程”，重点抓好现代物流、科技服务、信息服务、金融服务、商务服务、职业教育与培训等生产性服务业，改造提升商贸服务、餐饮服务、房地产业、家庭及社区服务等生活性服务业，着力培育节能环保、工业设计、健康服务、服务外包、会展服务、汽车服务、总部经济等新兴服务业，积极发展农村服务业，促进区域城乡服务业协调发展。三是进一步扩大消费需求。认真落实上级增加收入鼓励消费的政策措施，增强居民消费能力。大力培育新的消费热点，拓宽消费领域，推动消费结构优化升级。加强和改善对房地产市场的调控，增加有效供给。完善农村流通网络建设，扎实推进“万村千乡”市场工程，加快大型连锁超市与农产品质量安全示范区“区超对接”，推进农产品进城、工业品下乡。深入开展“满意消费惠万家”和诚信商贸企业创建活动，严厉打击侵犯知识产权和制售假冒伪劣商品等商业欺诈行为，不断改善消费环境。

　 (四)坚持以招商引资、项目建设为抓手，培育和增强经济发展的后劲。项目建设特别是大项目建设是转方式调结构的重要载体，是经济发展的根本支撑，必须坚持不懈地抓在手上、抓出成效。一是抓好重点项目建设。围绕强市名城建设目标要求，抓好100个重点建设项目，力争完成投资 450亿元。着力抓好中国重汽工业园、青年汽车二期、航天特车扩能、550千伏SF6断路器及特变电工华东科技园等一批优势产业项目建设，着力打造区域特色；着力抓好华兴纺织年产2000吨壳聚糖纤维产业化项目、山东泰邦生物制品扩能改造等一批战略性新兴产业项目，加快形成新的增长极；着力抓好瑞星集团“3052”工程、阿斯德化工100万吨/年控释肥、蒙牛乳业低温奶加工生产线建设项目等一批传统产业项目建设，努力实现传统产业改造升级。同时，加大项目策划、储备力度，积极谋划和储备一批有利于我市推进结构调整、保障和改善民生的重大基础设施和重大产业项目，策划总投资过亿元项目80个。二是努力扩大招商引资。创新体制机制，创新招商方式，突出产业招商，在招大引强上下功夫、求突破。发挥企业招商的主体作用，积极推进委托招商、以商招商、园区招商、会展招商、境外招商等。认真研究后金融危机时代产业转移和资本流动的规律，围绕我市优势产业，谋划组织好一些高层次的招商活动，突出战略性新兴产业和高端制造、现代服务业、特色农业，有针对性地引进一些大企业、大集团、大项目，提高利用外资的质量和档次。三是抓好对上争取。加大对上争取力度，争取更多的项目与资金落户泰安。实施好中央扩大内需项目和省调控资金项目，落实好配套资金和建设条件，加快项目建设进度，确保工程质量和资金安全，确保在建和续建项目如期顺利完成。四是抓好投资结构调整。切实发挥财政投资对结构调整的引导作用，优先保证重点在建、续建项目的资金需求。坚持区别对待、分类指导，引导投资进一步向重点行业、关键领域和薄弱环节倾斜，依靠投资结构优化产业结构。今年计划对社会民生、高新技术和战略性新兴产业、现代服务业、技术改造、农林水利等八个关键领域投资1290亿元左右，约占规模以上投资的85%左右。严格执行投资项目用地、节能、环保、安全等准入标准，提高投资质量和效益。严格控制“两高”行业投资过快增长，年内不审批、核准、备案“两高”和产能过剩行业扩大产能项目。五是协调京沪高铁、山西中南部铁路通道、东平湖至济宁段调水与航运工程等国家、省重大基础设施建设，推进一批重大项目前期工作。

　 (五)坚持以“创城”为引领，加快推进城镇化进程。围绕强市名城建设目标，以争创“中国人居环境奖”为动力，加快推进城乡一体化和城市现代化，全面提升城市综合承载力和竞争力。一是强化规划的龙头和调控作用。进一步提高规划的科学性、前瞻性、特色性，积极开展城市群和跨区域的城镇体系规划研究，突出抓好重点片区、大型公建设施的规划，以城市控制性详细规划和村镇规划为重点统筹城乡规划，大力推进规划全覆盖。加大规划执法力度，确保规划的严肃性。二是集中力量抓好“创城”大项目建设。加快推进中青旅泰山龙曦国际旅游度假地、宋庆龄基金会(泰山)海外华人休养中心、国际动态会展城、名嘉城市广场、报业文化产业园、瑞奥不夜城、盐海神汤国际会议中心等项目建设。开工建设奥特莱斯现代服务产业园、中华盛世文化博览园、泰安文化艺术中心等项目，以项目建设提升“创城”成果，提升城市形象和品位。三是继续抓好基础设施配套完善。继续推进“两轴”和“两带”片区、旅游经济开发区、泰山景区、高铁新客站片区、长城路片区等基础设施和配套建设，加快实施大汶河综合开发后续建设项目，加快泰安中心客运站建设和青兰高速及104国道绕城线改建。完善城市道路、园林绿化、生活垃圾无害化处理、污水处理等设施建设，重点抓好财源大街西段改造、灵山大街中段建设、梳洗河综合整治、城区东部天然气环网提压等工程建设，努力提升城市综合服务功能。大力实施公交优先发展战略，不断提高公交出行分担率，努力缓解泰城交通拥堵的压力。四是加快推进城镇化进程。在强化泰城中心城市地位的同时，加快膨胀县城，提升发展质量和水平，增强承载能力。加快发展城市周边城镇、大型工矿业所在地城镇、交通干道沿线城镇，通过扩容提质，强化产业和人口聚集，形成特色鲜明、功能完善的小城镇体系，突出抓好4个国家重点镇和15个省级中心镇建设。稳妥有序推进农村新型社区建设，搞好城中村改造，加快城乡一体化进程。五是着力创新体制机制。探索建立现代化城市管理长效机制，努力实现城市管理工作制度化、规范化、常态化。按照“整合资源、多方融资、自求平衡、滚动发展”的要求，规范和利用好融资平台，提高市场化融资能力，为城镇化提供资金保障。认真贯彻“三公八制”原则，加强从项目规划设计到竣工验收全过程程序化操作、规范化运作和严格化监管，提高政府投融资绩效。

　 (六)坚持改革开放，增强发展的动力和活力。坚持市场化改革取向，突出抓好重点领域和关键环节改革。一是继续推进国有企业改革。按照“先急后缓、方式多样、重点突出、分类实施”的原则，分情况分批次推进国有企业改革改制进程，健全完善现代企业制度，解决改制企业遗留问题。完善国有资产管理体制机制，调整优化国有经济布局结构，提高国有经济竞争力。二是深化农村改革。开展农村土地承包经营权、水域使用权等确权登记工作，制定完善鼓励土地承包经营权流转政策，积极稳妥地推进农村土地承包经营权流转市场建设，提高农民土地流转效益和土地经营规模效益。年内全面完成集体林权制度主体改革任务，加快集体林改配套改革，深化农村金融和农村综合配套改革。完成在全市普遍建立健全乡镇或区域性农业技术推广、动植物疫病防控、农产品质量监管等公共服务机构改革任务。三是继续深化医药卫生体制改革。总结新泰、宁阳试点经验，在全市范围推开基本药物制度。全面落实医改实施方案，确保达到上级要求的医改进度。四是深化价格改革。按照国家、省的统一部署，稳步实施水、电、热等资源性产品价格和环保收费改革。审慎出台调价方案，充分考虑社会承受能力，完善配套措施。五是深化财税体制改革。完善财政转移支付制度，健全县级基本财力保障机制。健全政府预算体系，将各类政府性收入全部纳入预算管理。规范政府投融资平台运行，加强政府债务管理，防范债务风险。同时，深化事业单位人事制度改革，建立符合事业单位特点的管理制度。

　 坚持两个市场、两种资源并重，出口与进口、“引进来”和“走出去”并举，不断提高外向型经济发展水平。在抓好招商引资的同时，创新机制，用好跨境贸易人民币结算、出口信用担保等政策，支持和鼓励扩大出口，重点培植40家出口企业群体。紧紧依托我市主导产业和骨干企业，鼓励引导转移产能和承包境外工程，大力发展服务贸易和服务外包，规范外派劳务市场秩序。鼓励企业通过增资扩股、资产重组、联合购并等方式合资与合作。支持优势企业境内外上市，提高融资能力。加强区域经济合作，积极融入省会城市群经济圈发展，加强与国内外企业推进基础设施、产业、市场、社会管理等方面的融合发展。充分利用京沪高铁开通的效应，加强与沿线城市的联系，主动承接发达地区产业转移。扎实做好新一轮对口支援新疆岳普湖县、扶贫协作重庆巫溪县等项工作。

　 (七)坚持创新驱动，加快建设资源节约型和环境友好型社会。一是大力推进自主创新。充分发挥政府的主导作用、企业的主体作用、科研机构和大专院校的引领作用，切实搞好产学研紧密结合的科技创新体系建设。加强科技创新平台建设，力争更多的创新平台成为国家级创新平台。进一步完善科技投入机制，有效整合各类科技资源，认真落实国家支持企业创新的各项政策，鼓励企业加大研发投入，培育更多的创新型企业，力争年内国家级高新技术企业达到60家。年内实施技术创新计划1000项，其中省级以上220项。实施质量兴市战略，加强商标、专利、标准化、名牌工作，培植一批名牌企业和产品。全面实施《泰安市中长期人才发展规划纲要》，强化政策服务，加强各类人才队伍建设，为发展高端高质高效产业提供强有力的人才支持。二是持续推进节能减排。坚持两手并重，一手抓淘汰落后产能，一手抓源头控制，强化目标、落实责任，确保完成省政府下达的节能减排任务。严格执行高耗能企业限制产能计划，坚决淘汰高耗能高污染行业及过剩落后产能，继续抓好土小生产线关停，突出抓好9大高耗能行业和耗煤5000吨以上企业、电力负荷1万千瓦以上企业的节能管理。抓好重点区域、重点领域的节能工作，推进10大重点节能工程，突出抓好100户重点用能企业。实行节地、节能、节水、节材，大力发展绿色经济、低碳经济、循环经济，推进节能减排。三是加强生态环境建设。实施《泰安生态市建设总体规划》，以争创国家环保模范城市为动力，坚持污染防治与生态保护并重，强化水、大气、土壤、固体废物等污染治理，实施造林绿化、湿地保护、水土保持、农业面源污染控制、破损山体治理、环境综合治理等保护治理自然生态工程，健全完善环境资源补偿机制和考核奖惩制度，建立起经济发展与资源节约、环境保护、生态建设相互促进的发展机制，改善城乡人居环境，促进人与自然和谐发展。

　 (八)坚持民生优先，促进社会繁荣和谐进步。坚持把保障和改善民生作为加快转变经济发展方式的根本目的，以实事落实为抓手，完善民生政策，加强社会建设，推进基本公共服务均等化。一是千方百计稳定和扩大就业。继续实施更加积极的就业政策，坚持就业优先发展战略，多渠道开发就业岗位，重点做好高校毕业生、农村转移劳动力、城镇就业困难人员和退役军人的就业安置工作。大力实施全民创业战略，在创业带动就业上实现新突破。有针对性地开展“4050”人员、“双零”家庭等困难人员就业援助工作。建立统一规范的人力资源市场，完善公共就业服务体系。二是加快完善社会保障体系。宣传贯彻《社会保险法》，完善各项社会保险制度和市级统筹办法，不断加大社会保险扩面征缴力度，年内城镇职工养老保险、城镇居民基本医疗保险覆盖率达到 98%以上，养老、医疗、失业等项社会保险费收缴率达到95%以上，调整提高企业退休人员养老金。扎实做好泰山区和新泰市国家级和省级新农保试点工作，积极开展城镇非就业居民基本养老保险试点，逐步建立覆盖城乡居民的社会保障体系。将新农合、城镇居民基本医疗保险政府补助标准统一提高到200元，提高城乡居民人均基本公共卫生服务经费。城乡低保月人均补助水平提高幅度不低于15元，农村低保标准提高到每人每年1400元。探索建立五保供养标准自然增长机制，建立孤儿基本生活保障制度，提高散居孤儿的养育标准，加快市、县(市区)社会福利服务中心建设。三是加快推进保障性住房建设。强化政府责任，多渠道增加保障性住房投入，加大公租房等保障性安居工程建设力度。今年住房公积金缴存比例由7%提高到8%。市级新开工建设公共租赁住房、廉租住房和经济适用房均有较多增加。四是大力发展社会事业。全面实施《泰安市中长期教育改革和发展行动计划》，加大投入，普及学前一年教育，均衡发展义务教育，全面推进素质教育，完善发展高中段教育和职业技术教育、成人教育，支持驻泰高校的改革与发展。大力实施中小学校舍安全工程建设，年内完成加固、重建校舍面积40万平方米。完善城乡医疗卫生、疾病控制、健康教育、妇幼保健、应急救治等公共卫生服务体系，为群众提供满意的基本医疗卫生服务。加快卫生基础设施建设，年内市中医院新病房楼、市精神病医院门诊病房楼力争投入使用。大力开展爱国卫生运动，完成农村改厕 1.9万座。继续深化文化体制改革，加快完善公共文化服务体系，继续做好20户以下自然村的广播电视“村村通”、文化资源信息共享、农家书屋和农村电影放映等工作，扎实做好第十届中国艺术节筹备工作，搞好文化遗产保护和利用。强化人口与计划生育目标管理责任制，推进人口信息平台建设，切实稳定低生育水平。加强体育场馆等基础设施建设，广泛开展全民健身活动，提升竞技体育水平。依法加强宗教事务管理，认真落实少数民族政策，扶持发展少数民族经济。加强民主法制建设，完善基层民主管理制度。深化社会主义核心价值体系建设，大力推进以文明城市创建为龙头的群众性精神文明创建活动、公民思想道德建设、青少年思想道德建设。重视抓好国防动员、人民防空、民兵预备役、双拥共建工作，积极争创新一轮国家双拥模范城。关心支持老龄、妇女儿童、残疾人事业发展，做好外事侨务和史志、档案、气象、防震减灾等项工作。五是加强和创新社会管理。以我市被列为“全国社会管理创新综合试点城市”为契机，逐步健全基层社会管理和服务体系及应急管理体制机制，提高社会管理服务和应突处置能力。进一步完善社会治安综合治理防控体系，加大社会管理薄弱环节整治力度，依法防范和严厉打击各类违法犯罪活动，切实保障人民生命财产安全。深入实施“安全生产基层基础深化年”活动，加强对矿山、化工、交通、森林、公共场所等重点行业、重点领域的安全生产专项整治，坚决遏制重特大安全事故。强化重大疫病防控和食品药品安全监管。高度重视人民来信来访，畅通群众利益诉求表达渠道，有效化解各种不稳定因素，维护社会和谐安定。

　 四、加快政府职能转变，努力建设人民满意政府

　 新形势、新任务对政府工作提出了新的更高要求。要以加快职能转变为核心，进一步提高施政能力和水平，努力建设为民、务实、清廉、人民信赖的政府。

　 (一)建设学习型政府。政府工作人员要牢固树立终身学习的理念，勤于学习、善于思考，全面系统地学习中国特色社会主义理论体系，重视加强对科学发展观、转方式调结构等重大理论和决策的学习，不断增强深入贯彻落实科学发展观的自觉性和坚定性。重视优化知识结构，加强履行岗位职责所需要的专业知识和相关领域知识的学习，深入调查研究，拓宽视野思路，不断提升发展境界。坚持理论联系实际，学以致用，以用促学，努力提高科学判断形势、驾驭复杂局面、解决实际问题的能力和水平，以此推进各项工作取得新突破。

　 (二)建设服务型政府。服务人民、服务社会、服务发展是各级政府的神圣职责。牢固树立为民执政理念，把党和人民赋予的职责看得比泰山还重，带着觉悟、带着责任、带着感情真心实意为人民群众办实事、解难题、谋实利。适应市场经济发展的需要，加快转变政府职能，强化经济调节、市场监管、社会管理和公共服务，为科学发展营造良好环境。政府及其组成部门要进一步改进服务方式，简化办事程序，规范行政行为，提高工作效能。大力加强作风建设，以“创先争优争做泰山先锋”活动为契机，把高度的工作热情和求真务实的作风结合起来，始终保持良好的精神状态，创新思维，勇于担当，永争一流，对干事创业充满旺盛的激情，对人民群众充满浓厚的感情，全身心投入到各项工作中。进一步完善考核评价体系，改进考核方式，引导各级更加重视质量效益，形成有利于科学发展的工作导向。把狠抓工作落实作为转变作风的关键环节，用更多的精力、更大的力度狠抓任务落实、责任落实，强化调度督查，确保各项工作落到实处、见到实效。

　 (三)建设法治型政府。依法行政是建设法治政府的核心内容，也是政府行为的基本准则。认真做好“六五”普法和“五五”依法治市规划的调研起草和启动实施，提高全社会自觉学法、守法、用法意识，夯实社会和谐的法治基础。政府工作人员要进一步强化法制意识，深入贯彻落实国务院、省政府关于推进依法行政的决策部署，加强行政执法队伍建设，严格规范公正文明执法，不断提高行政执法水平。坚持依法科学民主决策，加快建立健全决策、执行、监督相互制约、协调运转的行政运行机制，做到行政合法、程序正当、高效便民、诚实守信、权责统一。深入贯彻落实行政监察法、领导干部经济责任审计规定等法律法规，加快推进行政问责制度化、规范化进程，切实做到有权必有责、用权受监督、违法要追究。坚持向人大报告工作和向政协通报工作制度，认真执行人大及其常委会的决议、决定，自觉接受人大的法律监督和政协的民主监督及社会舆论监督，认真办理人大代表议案、建议和政协提案。加强同各民主党派、工商联、人民团体和社会各界人士的联系。

　 (四)建设阳光型政府。深入贯彻实施政府信息公开条例，进一步推进政务公开，让政府权力在阳光下运行。大力推行电子政务，加强市政府门户网站、公共行政服务中心、便民服务电话受理中心等服务窗口建设，不断完善政务服务网络，全面推行行政服务标准化，切实提升公共服务水平。进一步健全完善政府决策、专家咨询、民主听证、社会公示等各类公开办事制度，充分保障人民群众的知情权、参与权和监督权，努力提高政府工作的透明度和公信力。进一步加强惩治和预防腐败体系建设，加大查办违纪违法案件力度，重点加强对主要领导干部、关键岗位人员和财政资金使用、土地使用权出让、工程招投标等重点领域、重要环节的审计与监督。深化政风行风民主评议、行风热线等工作，加强对群众反映强烈的突出问题的专项治理，着力解决发生在群众身边的腐败问题。认真贯彻落实新颁布的《关于实行党风廉政建设责任制的规定》，模范遵守领导干部廉洁自律的各项规定，严于律己，带好队伍，树立清正廉洁的良好形象。

　 各位代表！新的形势鼓舞人心，新的蓝图催人奋进。让我们紧密团结在以胡锦涛同志为总书记的党中央周围，全面贯彻党的十七大和十七届五中全会精神，深入贯彻落实科学发展观，在市委的坚强领导下，万众一心，真抓实干，锐意进取，奋力拼搏，为圆满完成“十二五”规划确定的宏伟目标，加快推进强市名城建设、全面建设小康社会而努力奋斗！
